# Uncovering Molecular Bases Underlying Bone Morphogenetic Protein Receptor Inhibitor Selectivity

**DOI:** 10.1371/journal.pone.0132221

**Published:** 2015-07-02

**Authors:** Abdelaziz Alsamarah, Alecander E. LaCuran, Peter Oelschlaeger, Jijun Hao, Yun Luo

**Affiliations:** 1 Department of Pharmaceutical Sciences, College of Pharmacy, Western University of Health Sciences, Pomona, California, United States of America; 2 College of Veterinary Medicine, Western University of Health Sciences, Pomona, California, United States of America; University of Akron, UNITED STATES

## Abstract

Abnormal alteration of bone morphogenetic protein (BMP) signaling is implicated in many types of diseases including cancer and heterotopic ossifications. Hence, small molecules targeting BMP type I receptors (BMPRI) to interrupt BMP signaling are believed to be an effective approach to treat these diseases. However, lack of understanding of the molecular determinants responsible for the binding selectivity of current BMP inhibitors has been a big hindrance to the development of BMP inhibitors for clinical use. To address this issue, we carried out *in silico* experiments to test whether computational methods can reproduce and explain the high selectivity of a small molecule BMP inhibitor DMH1 on BMPRI kinase ALK2 *vs*. the closely related TGF-β type I receptor kinase ALK5 and vascular endothelial growth factor receptor type 2 (VEGFR2) tyrosine kinase. We found that, while the rigid docking method used here gave nearly identical binding affinity scores among the three kinases; free energy perturbation coupled with Hamiltonian replica-exchange molecular dynamics (FEP/H-REMD) simulations reproduced the absolute binding free energies in excellent agreement with experimental data. Furthermore, the binding poses identified by FEP/H-REMD led to a quantitative analysis of physical/chemical determinants governing DMH1 selectivity. The current work illustrates that small changes in the binding site residue type (e.g. pre-hinge region in ALK2 *vs*. ALK5) or side chain orientation (e.g. Tyr219 in caALK2 *vs*. wtALK2), as well as a subtle structural modification on the ligand (e.g. DMH1 *vs*. LDN193189) will cause distinct binding profiles and selectivity among BMP inhibitors. Therefore, the current computational approach represents a new way of investigating BMP inhibitors. Our results provide critical information for designing exclusively selective BMP inhibitors for the development of effective pharmacotherapy for diseases caused by aberrant BMP signaling.

## Introduction

The bone morphogenetic proteins (BMPs), a subgroup of the transforming growth factor-β (TGF-β) superfamily, play critical and diverse roles in cellular processes [1]. The biological activities of BMPs are mediated through formation of heteromeric BMP receptor complexes consisting of two type I receptors and two type II receptors. When BMPs bind to the extracellular part of the receptor complex, the type I BMP receptors are activated and their intracellular kinase domain then phosphorylates R-Smads protein family to trigger downstream gene transcription [2]. A series of serine/threonine kinases, termed activin receptor-like kinases (ALK), have been identified to constitute BMP type I receptors (BMPRIs): ALK1, ALK2, ALK3, and ALK6.

Aberrant activation of BMP signaling is involved in numerous diseases and targeting BMPRIs is believed to be an effective therapeutic approach for treating these diseases. For instance, mutation R206H in ALK2, which constitutively activates BMP signaling in the absence of BMP ligands, is responsible for ~97% of patients with fibrodysplasia ossificans progressiva (FOP) disorder, one of the most devastating and rare bone diseases [3, 4]. Thus small molecular ALK2 inhibitors, which may be effective therapeutic agents against FOP, have been highly sought after. In addition, abundant expression of ALK1 was found in the vasculature of many types of tumors, but weak or no expression of ALK1 was detected in tumor cells and normal tissues, suggesting that ALK1 inhibition may be a potential therapeutic approach complementary to the current anti-angiogenic modalities in the clinic [5]. Similarly, ALK3 and ALK6 are also implicated in other distinct diseases [6–8]. Therefore, development of selective small molecule inhibitors of each subtype of BMPRIs to block BMP signaling may represent an effective therapeutic approach to treat these different types of disease.

**Fig 1:**
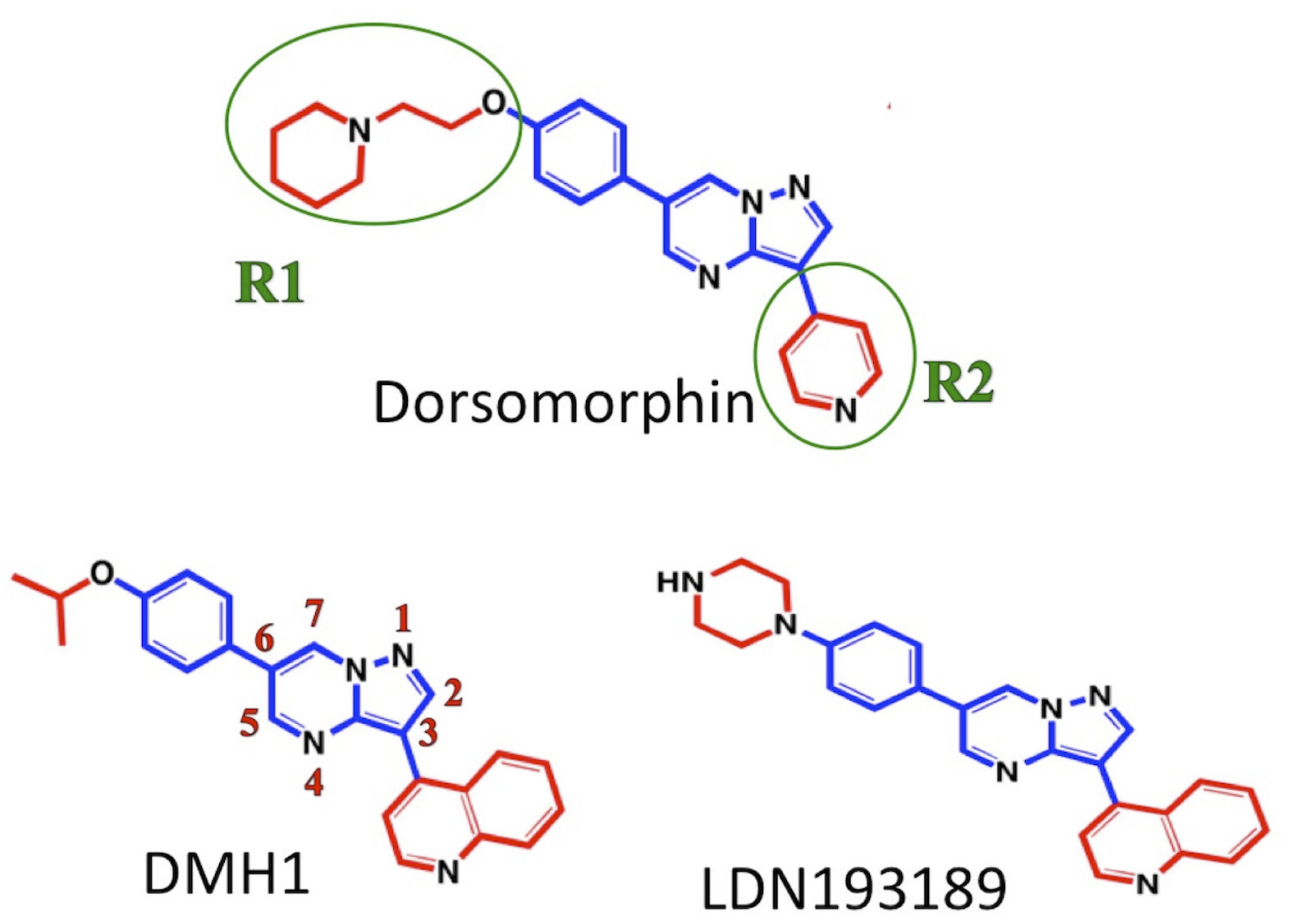
Structures of dorsomorphin and its analogs. The common scaffold is shown in blue, R1 and R2 groups are shown in red.

Recently, significant efforts have been made to develop small molecule ALK2 inhibitors to interrupt abnormal activation of BMP signaling. Dorsomorphin (Fig 1), the first small molecule BMPRI inhibitor, was identified in a screen for compounds that perturb the zebrafish embryonic dorsoventral axis [9]. Although dorsomorphin inhibits ALK2 activity by binding to the ATP-binding pocket of the ALK2 Ser/Thr kinase domain [10], it displays significant “off-target” inhibition of the vascular endothelial growth factor receptor type 2 (VEGFR-2) tyrosine kinase and other BMP type I receptors [11–13]. Over the past several years, a series of dorsomorphin analogs with pyrazolo[1,5-**a**]pyrimidine or aminopyridine scaffold have been developed to improve compound selectivity towards ALK2 [13–20] (S1 Table). For instance, DMH1 [13] was developed with higher selectivity towards BMP type I receptors *vs*. TGF-β/Activin pathway receptor ALK5 and VEGFR2 than dorsomorphin. Other derivatives such as LDN-193189 [16, 21], exhibited higher potency against BMP type I receptors but less selectivity against ALK5 and VEGFR2 than DMH1 (Fig 1 and S1 Table). Despite the continuous efforts in chemical synthesis in recent years, it remains unclear how these BMP inhibitors can discriminate one receptor over others. A consensus has emerged that understanding the selectivity mechanisms is critical for designing exclusively selective inhibitors for each subtype of BMPRIs that are urgently needed today.

To address this question, we apply all-atom molecular dynamics-based free energy calculations to investigate the physicochemical contributions underlying BMP inhibitors’ binding characteristics, which are often difficult to obtain from ligand-based structure-activity relationship (SAR) analysis or static crystal structures. The main computational approach applied here is free energy perturbation coupled with Hamiltonian replica-exchange molecular dynamics (FEP/H-REMD) simulations. The FEP/H-REMD approach has recently provided a wealth of molecular details on the energetic determinants of the binding affinity in tyrosine kinases [22–25]. We have chosen DMH1 as a model compound with the aim of capturing the origin of its excellent selectivity towards ALK2 *vs*. the structurally closely related ALK5 and VEGFR2 kinases.

For ALK2 kinase, two crystal structures were used for this study. One is from the wild-type ALK2-dorsomorhin complex (PDB ID: 3H9R [10]), denoted as wtALK2. The other is that of the Q207D mutant ALK2-LDN193189 complex (PDB ID: 3Q4U [20]). The mutation Q207D is located at the GS domain (glycine-serine rich region) on top of the kinase N-lobe (N-terminal lobe) motif (S1A Fig). Q207D ALK2 has been reported as constitutively active ALK2 (caALK2); it leads to ectopic endochondral bone formation in a mouse model [16]. ALK2 and ALK5 are highly similar (S1B Fig for structural alignment result), and their kinase domains contain a conserved sequence of three amino acids (Asp-Leu-Gly) known as DLG-motif at the beginning of the activation loop (A-loop), while VEGFR2 tyrosine kinase contains a DFG motif (Asp-Phe-Gly). In general, the crystal structures of ALK2 and ALK5 with inhibitors all represent DLG-in like conformation (S1C Fig) [10, 20, 26], while in VEGFR2, there are various inhibitors bound to either the DFG-in or DFG-out conformation (sometimes denoted as type-I and type-II inhibitors). Therefore, we have chosen crystal structures of both DFG-in and DFG-out conformations of VEGFR2 (indicated as VEGFR2-in and VEGFR2-out) as separate topologies for DMH1 binding free energy calculation.

The results show that, while molecular docking method used here gave nearly identical scores among the three kinases, FEP/H-REMD simulations successfully reproduced that DMH1 only binds to ALK2, but not to ALK5, VEGFR2-in or VEGFR2-out. The binding free energies are in good agreement with experimental measurements (Table 1). The FEP/H-REMD identified the potential binding poses of DMH1, which led to the quantitative analysis of the origin of DMH1 selectivity for these kinases. Our calculations indicate that DMH1 selectivity originates from a favorable electrostatic interaction between DMH1 and the ATP-binding pocket of ALK2. This interaction is absent in ALK5 and VEGFR2 because of subtle binding pose changes. Confirming our computational predictions, we further elucidate that the compound LDN193189 has more favorable interaction with ALK5 than DMH1, which is consistent with previous experimental reports [14, 19]. Our computational study highlights the importance of structural dynamics and demonstrates that the FEP/H-REMD approach can serve as a robust method to explain and predict binding selectivities of BMP inhibitors among highly conserved ATP binding sites. The molecular mechanism illustrated here provides critical information for future rational design of exclusively selective and potent inhibitors for each subtype of BMPRIs.

**Table 1:** Calculated binding free energy $\Delta G_{b}^{o}$ of DHM1 to different kinases, docking scores and results from experimental kinase assays in kcal/mol.

*Protein*	*Expt* ^a^	*Docking score* ^b^	$\Delta G_{b}^{o}$ ^c^ from FEP/HREMD	*Decomposition* ^*d*^ *of $\Delta G_{b}^{o}$*			
				repu	disp	elec	rst
**wtALK2**	-9.6	-8.9 ± 0.1	-8.5± 0.6	12.19	-17.65	-3.16	0.08
**caALK2**	-9.6	-9.6 ± 0.1	-6.2± 0.4	14.41	-18.93	-1.73	0.02
**ALK5 pose3** *	>-5.5	-9.1 ± 0.1	-0.4± 0.7	7.48	-15.86	7.42	0.55
**ALK5 pose4**	>-5.5	-8.7 ± 0.1	-0.9± 0.7	9.76	-16.16	4.78	0.68
**VEGFR2-in**	>-5.5	-8.6 ± 0.2	-3.4± 0.6	10.29	-18.91	4.63	0.60
**VEGFR2-out**	>-5.5	-9.7 ± 0.1	-1.7± 0.6	9.67	-15.54	3.66	0.49

^*a*.^ The experimental binding free energies were estimated from IC_50_ of kinase assay [14] using Δ*G = -RTln*(C°/*K*_*d*_), in which *R* is the gas constant 1.987×10^−3^ kcal/K/mol, *C°* is the standard reference concentration 1 mol/L, and *T* is 300 K.

^***b***.^ The docking score is a cluster average value. Errors in docking scores are standard deviations from all binding poses within a single cluster.

^*c*.^ The average and the standard deviations in $\Delta G_{b}^{o}$ are calculated from the last five simulations of 400 ps per replica with different initial velocities.

^***d***.^ The free energy decomposition gives repulsive, dispersive, electrostatic, and distance restraint contributions in the total $\Delta G_{b}^{o}$.

**Fig 2:**
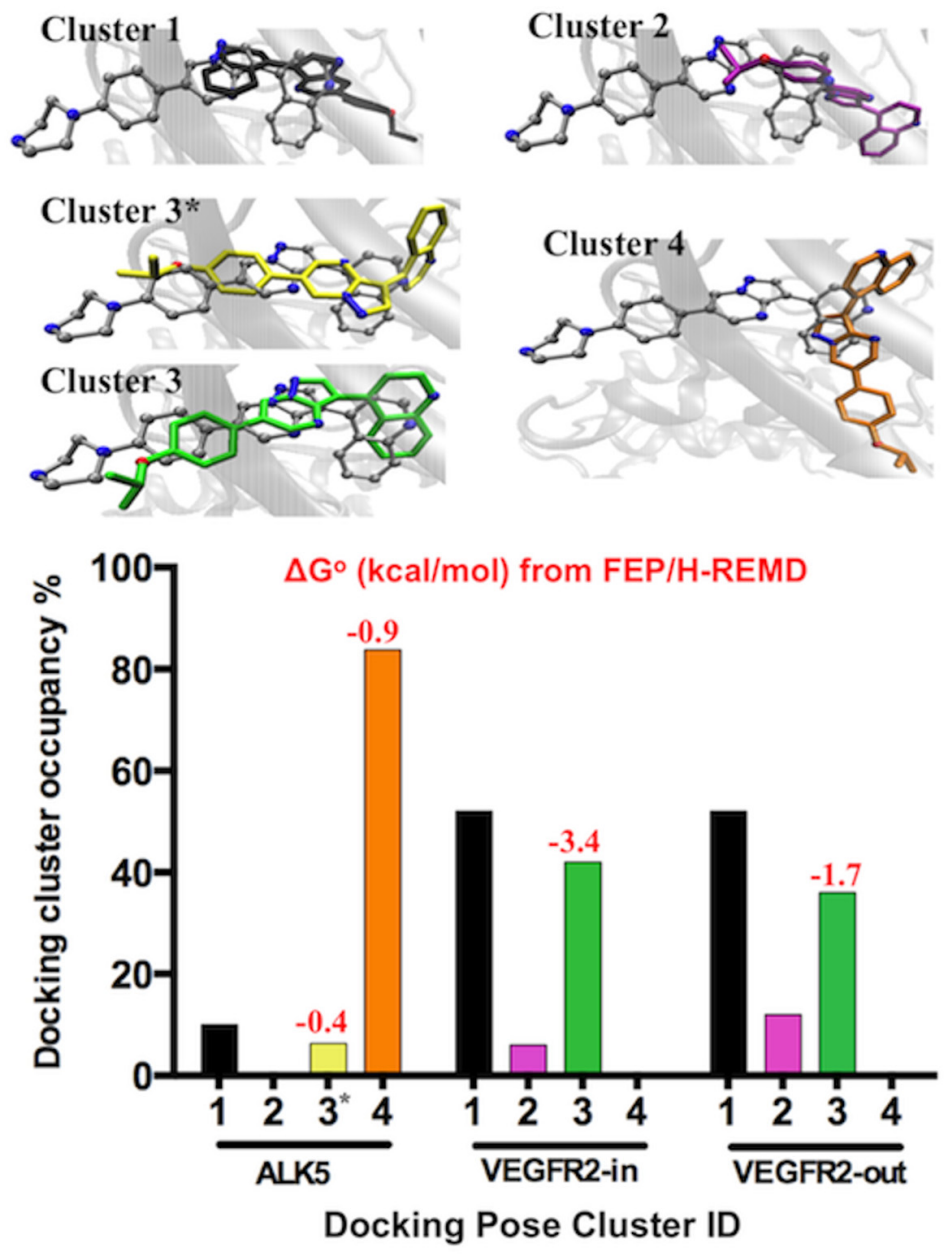
Clustered DMH1 docking poses in ALK5, VEGFR2-in, and VEGFR2-out proteins. Each docking cluster represents a group of ligand poses with ligand RMSD < 2 Å. **Top:** Representative docking poses in each cluster superimposed on LDN193189 (gray) in the ALK2 x-ray structure. **Bottom:** The occupancy of each docking cluster, with FEP/H-REMD binding free energy shown in red on top of each histogram (positive values are not shown here). The color of the ligand-binding pose corresponds to those in the bar graphs in the bottom.

* The pose 3* in Fig 2.

## Materials and Methods

### Atomic model preparation

The initial structures of ALK2, ALK5 and VEGFR2 for simulations were taken from the crystal structures of the protein-inhibitor complexes (PDB ID: 3H9R for wtALK2 [10], PDB ID: 3Q4U for caALK2 [20], PDB ID: 3TZM for ALK5 [26], PDB ID: 3VO3 for VEGFR2 DFG-out [27], PDB ID: 3CJG for VEGFR2 DFG-in [28]) of *Homo sapiens* species. In the wtALK2 complex, part of the A-loop (residues 362 to 374), and the β-turn between β4 and β5 (residues 273 to 275) were not present in the crystal structure. To address this issue, the missing A-loop portion in wtALK2 was transplanted from the crystal structure of the constitutively active Q207D mutant ALK2 (caALK2). The three missing residues in the β-turn were patched using the PATCH command in CHARMM program [29, 30]. Then these patched residues underwent energy minimization with the rest of the protein fixed to optimize the conformation. The pKa calculations using PROPKA GUI [31] plugin in VMD [32] indicate that the ionization states of protein residues remain the same as that of the individual residues at physiological pH. All the crystal water molecules were kept unchanged. CHARMM-GUI [33] was used to read in the PDB files and solvate each system in a rectangular water box (94 Å × 94 Å × 76 Å). Since potassium and chloride ions are the two major cytosolic ions, each system was neutralized with K^+^ and Cl^-^ ions at a physiological salt concentration of 150 mM. The solvated DMH1 complexes with wtALK2, caALK2, ALK5, VEGFR2 DFG-in and VEGFR2 DFG-out consist of 53747, 53706, 68303, 67950 and 53824 atoms, respectively. All simulations employed the all-atom CHARMM C36 force field [34–36] for proteins and ions, and the TIP3P force field [37] for water. In addition, the missing partial P-loop (residues 843 to 846) and the partial A-loop (residues 1052 to 1065) in the crystal structure of VEGFR2 DFG-in were patched using the CHARMM PATCH command. Likewise, in ALK5, the A-loop residues 370 and 371 were patched using CHARMM. The patched residues were subjected to 500 steps of energy minimization using the steepest descent method [38], followed by 500 steps of minimization using the adopted-basis Newton-Raphson method [38], with the remaining parts of the protein held fixed using CHARMM.

Small ligands were first prepared and minimized using the ArgusLab program [39]. DMH1 is expected to be neutral in bulk solution. The unsubstituted N atom on the piperazine ring of LDN193189 is solvent-exposed in the binding site and is expected to be protonated in a physiological pH aqueous solution. Small ligand force field parameters were generated using the General Automated Atomic Model Parameterization (GAAMP) web server [40]. For DMH1 in bulk solution, the center-of-mass of the ligand was placed at the center of a cubic water box with a side length of 90 Å, resulting in a total of 49102 atoms. The solvated system was equilibrated for 1 nanosecond (ns) before being submitted to solvation free energy calculations.

### Docking and Solvation

In order to determine the potential binding poses of DMH1 in different kinases, the ligand was docked into the ATP binding site of each minimized crystal structure using the flexible ligand docking protocol in Autodock4.2 [41]. Using AutoGrid [42], the grid box was set to 70, 80, and 70 grid points, along the x, y and z-axis, with 0.375 Å grid spacing, centered on the ATP binding site. For conformational search, docking calculations were carried out using the Lamarckian genetic algorithm and default parameters. The docking protocol was first tested by docking dorsomorphin back into the ALK2 crystal structure and showed perfect alignment with the ALK2-dorsomorphin crystal structure (PDB ID: 3H9R). For DMH1 docking, the top 100 poses were sorted by their docking scores and clustered by root mean squared deviation (RMSD) of ligand heavy atoms that differ from each other within 2 Å.

The top ranked docking pose of each cluster was solvated in 150 mM KCl aqueous solution using CHARMM-GUI, and the molecular dynamics equilibrium (see simulation protocol below) was set to relax the atomic system by releasing the harmonic constraints (force constant 50 kcal/mol/Å^2^) stepwise (every 200 ps) on water and ion molecules, protein side chains, protein backbone, and eventually the ligand. At least 40 ns of equilibration were carried out for each system without constraint before submitting for free energy calculation. RMSD of protein backbone and ligand heavy atoms as well as center-of-mass distance between protein and ligand were monitored to obtain stable binding poses (S2 Fig).

### Simulations Protocol

All the simulations were performed with NAMD2.9b [43] using periodic boundary conditions at constant temperature and pressure (NPT ensemble) of 300 K and 1 atm using Langevin thermostat and Andersen-Hoover barostat. Long-range electrostatics interactions were treated using the particle-mesh Ewald (PME) method [44]. The non-bonded interaction list was updated on every integration step using a cutoff of 13.5 Å. A smoothing function is applied to both electrostatics and van der Waals forces between 10 Å and 12 Å. The dynamics were propagated using Langevin dynamics with Langevin damping coefficient of 1 ps^-1^ and a time step of 2 fs. The SHAKE algorithm [45] was applied to all hydrogen atoms.

### Absolute Binding Free Energy Calculations

The binding of a ligand to a protein in aqueous solution can be described by the top part of the thermodynamic cycle in (S3 Fig). At equilibrium, the dissociation constant of the binding reaction is defined by $K_{d}\;=\;\frac{k_{off}}{k_{on}}\;=\;\frac{\left[Protein\right][Ligand]}{\left[Protein*Ligand\right]}$, which has the unit of concentration. Under the constant pressure and constant temperature ensemble (NPT), the Gibbs free energy of binding ΔG_bind_, i.e., the binding affinity, between receptor and ligand in solution is related to the dissociation constant *K*_*d*_ by Δ*G = -RTln*(C°/*K*_*d*_), in which *R* is the gas constant 1.987×10^−3^ kcal/K/mol, *C°* is the standard reference concentration 1 mol/L or 1 molecule/1660 Å^3^, and *T* is the absolute temperature in Kelvin. At room temperature, a nanomolar range dissociation constant corresponds to a binding free energy ΔG_bind_ range from -8 to -12 kcal/mol.

The free energy of binding can be estimated, in principle, from a long molecular dynamics trajectory, as long as the binding and unbinding events have occurred many times so as to give an accurate thermodynamic average. In practice, this brute-force approach is often hindered by the current computational limitations. Since the free energy is a function of state, the Free Energy Perturbation (FEP) approach [46, 47] can be used instead. In FEP, the bound and unbound states are connected through an arbitrary path by perturbing the Hamiltonian of the system in a series of alchemical steps. To calculate the absolute binding free energy using FEP, the double decoupling protocol developed by Deng and Roux [48] is applied. As illustrated in (S3 Fig), the total binding free energy is calculated from two separate steps: (1) a bound ligand is decoupled from its environment, i.e., the electrostatics and Lennard-Jones interactions between ligand and its environment (protein and solvent) are turned off gradually (ΔG_decouple_ step). This step corresponds to the free energy cost of moving a bound ligand from binding site into vacuum. (2) In order to close the thermodynamic cycle, the solvation free energy of ligand alone, i.e., the free energy cost of moving the ligand from vacuum to aqueous solution is calculated by decoupling the electrostatics and Lennard-Jones interactions between ligand and water molecules (ΔG_solvation_ step). The sum of the free energies of step (1) and step (2) equals the negative standard free energy of binding (ΔG_bind_ = -ΔG_decouple_- ΔG_solvation_) (S3 Fig).

The decoupling of a ligand with its environment (protein+solvent or solvent alone) was carried out using a scaling factor λ between 0 and 1, and a number of intermediate states. λ = 1 represents a fully interacting (bound) state, and λ = 0 represents a fully decoupled state (ligand in vacuum). The Weeks-Chandler-Andersen decoupling scheme [49] was utilized to separate the Lennard-Jones 6–12 potential into repulsive and dispersive parts. The decoupling of the ligand from the binding pockets was carried out as a stepwise reversible process, staged by the three thermodynamic coupling parameters representing repulsive, dispersive and electrostatic interactions of the ligand with its environment. Each coupling parameter has a different number of intermediate states between 0 and 1 using 128 λ in total (72 λ_dispersive_, 24 λ_repulsive_ and 32 λ_electrostatic_) or 64 λ in total (36 λ_dispersive_, 12 λ_repulsive_ and 16 λ_electrostatic_). In addition, a harmonic distance restraint between the center of mass of the ligand and center of mass of the protein is introduced using a force constant of 10 kcal/mol/Å^2^ to confine the sampling volume of the decoupled ligand. The free energy cost of introducing the distance restraint to the total binding free energy is calculated using equation $\Delta \Delta G_{t}\;=\;-k_{B}Tln\left(F_{t}C^{0}\right)-\Delta G_{t}^{site}$, where *F*_*t*_ corresponds to numerical integrals over the restraining quadratic potential and *C°* is the standard reference concentration. $\Delta G_{t}^{site}$ is the free energy cost of introducing the distance restraint when the ligand is fully coupled with the receptor. $\Delta G_{t}^{site}$ is calculated by decreasing the force constant stepwise (13 windows and 200 ps per window) from its original value to zero using the thermodynamic integration framework within the *colvars* module in NAMD2.9b. The contributions of the distance restraint are quite small (see Table 1), which indicates that all the reference distances are taken from well-equilibrated binding poses.

To further improve Boltzmann sampling, the Hamiltonian replica-exchange algorithm was coupled to FEP (FEP/H-REMD) [50–52]. The FEP/H-REMD method has been recently used to predict binding affinities of small molecular ligands to tyrosine kinases [23], oligosaccharide binding to glycoside hydrolase [53], as well as the binding of flexible decapeptide to SH3 domain of Abl kinase [25]. In this method, each λ value represents one replica of the system and frequent attempted λ-swapping (0.2 ps^-1^) between neighboring replicas was performed during the simulation. The attempted exchange was either rejected or accepted according to the Metropolis Monte Carlo criterion to ensure the detailed balance condition [54]. The average acceptance ratio among 128 replicas was > 80%. During the post-processing, the shuffled trajectories due to λ-swapping were un-shuffled for each λ value using the *sortreplicas* program in NAMD2.9b. The shuffled energy outputs were sorted for the final WHAM [55] post-processing. The insertion of the ligand into the bulk phases was calculated with the same protocol using 64 replicas in total. For each FEP/H-REMD job, all replicas were running in parallel mode on 512 or 1024 compute nodes on the IBM Blue Gene/Q supercomputer Mira at Argonne National Laboratory.

### Statistical Analysis

Paired T-tests for the root of mean standard fluctuation (RMSF) in the A-loop and P-loop were used to quantify the magnitude of the change between the apo (unbound) and DMH1 bound states utilizing Prism6 software (GraphPad Software Inc., La Jolla, CA). The individual amino acids in each loop were paired, for example: D354 in the apo A-loop is paired to D354 in the DMH1 bound A-loop. All statistically different results are denoted by one or more asterisks; * = p < 0.05, ** = p < 0.01, and *** = p < 0.001.

## Results and Discussion

### Multiple docking poses

The top ranked binding pose of ALK2-DMH1 is in excellent agreement with crystal structures of the ALK2-dorsomorphin and ALK2-LDN193189 complexes. However, the docking scores failed to distinguish DMH1 as a binder *vs*. non-binder between ALK2, ALK5 and VEGFR2 (Table 1). The flexible ligand docking offered three clusters of binding poses in both ALK5 and VEGFR2-in/out with nearly identical docking scores as ALK2-DMH1 (Fig 2). These docking poses cannot be experimentally confirmed since there is no crystal structure of these DMH1 analogs binding to ALK5 or VEGFR2. Multiple binding poses are also more likely to occur in the case of weak binding. Therefore, the FEP/H-REMD simulations were carried out for the entire top ranked DMH1 binding poses from each cluster identified by AutoDock4.2 regardless of the binding scores. The free energy calculations revealed that some docking poses were false positives (positive binding free energy). Therefore we eliminated those poses. When there were multiple poses that gave negative binding free energies from FEP/H-REMD, we combined the binding free energies using equation (1) $\Delta G_{\;}^{o}\;=\;-k_{B}Tln\left[\sum_{i\neq j}exp(-\frac{\Delta G_{i\neq j\;}^{o}}{k_{B}T})\right]$, similarly to the strategy applied in relative free energy calculations [56, 57].

#### FEP/H-REMD results show only one out of three docking poses in VEGFR2 has negative binding free energy

The docking binding poses of DMH1 in VEGFR2-out were clustered into three groups (Fig 2). The FEP/H-REMD simulations yielded positive binding free energies of +2.0 and +1.7 kcal/mol for poses (1) and (2), respectively. Only pose (3) gave a small negative binding free energy of -1.7 kcal/mol. This is consistent with the fact that only cluster (3) has binding features similar to the ALK2- LDN193189 crystal structure (Fig 2 top). The small negative binding free energy agrees with experimental kinase selectivity data [14]. Same as in VEGFR2-in, only the ALK2-LDN193189 like binding pose (3) gave a negative binding free energy of -3.4 kcal/mol. Therefore, for both systems, we used pose (3) for further analysis.

#### FEP/H-REMD results show two docking poses in ALK5 have negative binding free energy

The dominant binding pose cluster (4) is unique in ALK5, characterized by the quinoline ring overlapping with the pyrazolo-pyrimidine moiety of LDN193189 in the crystal structure, and the rest of the molecule projects away from the ATP site (Fig 2). The DMH1 binding pose (3*) in ALK5 is similar to the pose (3) in VEGFR2-in/out, but shifted into a hydrophobic region adjacent to the ATP binding site with a different rotation of the pyrazolo[1,5-**a**]pyrimidine and quinoline rings. For FEP/H-REMD calculations, we excluded pose (1) since it was shown to be a false positive binding pose in VEGFR2. Both poses (3*) and (4) turned out to be weak binding poses with binding free energies of -0.4 and -0.9 kcal/mol. Combining the two negative binding free energies using equation (1) yielded the binding free energy of -1.1 kcal/mol, which agrees with the experimental result that DMH1 is a weak binder for ALK5 [14].

### Absolute binding free energies and decomposed contributions

The absolute binding free energy of wtALK2-DMH1 complex was computed from a total of 384 ns FEP/H-REMD sampling (6 ns per replica, 64 replicas). caALK2-DMH1 and VEGFR2-out-DMH1 complexes were sampled for a total of 358.4 ns each. The evolution of the calculated free energy over the sampling length indicates the progress towards convergence (S4A Fig). The progression of the free energy components (repulsive, dispersive and electrostatic) with respect to the coupling parameters (λ_rep_, λ_dis_ and λ_elec_) for DMH1 in ALK2 *versus* DMH1 in water is shown in (S4 Fig). The ALK5-DMH1 and VEGFR2-in-DMH1 systems were sampled only for 256 ns, as the evolution of the calculated free energies clearly shows that the binding affinities were already less negative than -5 kcal/mol. The data collected from the last 128 ns were used to compute the block average using a block size of 400 ps and standard error of the free energies of binding for DMH1 to ALK2s, yielding -8.5 ± 0.6 kcal/mol in wtALK2 and -6.2 ± 0.4 kcal/mol in caALK2. The data collected from the last 76.8 ns were used to compute free energies for the other systems, yielding -0.4 ± 0.7 kcal/mol and -0.9 ± 0.7 kcal/mol in ALK5 (see multiple docking poses section), -3.4 ± 0.6 kcal/mol in VEGFR-in, and -1.7 ± 0.6 kcal/mol in VEGFR-out (Table 1).

#### Electrostatic contribution is a key molecular determinant responsible for the binding specificity

The staged FEP/H-REMD strategy separates the interaction free energy of ligand with its surrounding into repulsive, dispersive and electrostatic components (Methods section). Although the absolute value of each decomposed free energy is path dependent, comparing the relative values between studied kinases offers useful insights into the binding mechanism. The positive repulsive contribution of the binding free energy in all proteins versus in bulk solution (Table 1) suggests that, in order to accommodate the bulky ligand DMH1, the binding pocket of all three kinases must undergo a certain amount of structural rearrangements, including certain numbers of water molecules expelled from the binding pocket and rearrangements of binding site residues. These rearrangements are associated with an unfavorable free energy penalty. The major favorable contribution of the binding affinity is the dispersive component. The negative dispersion contribution in protein relative to bulk solvent suggests that the protein binding site provides an environment with a higher density of van der Waals centers to stabilize DMH1 in the binding pocket. However, the dispersive component itself does not reflect the trend of the binding affinity among ALK2, ALK5 and VEGFR2, which indicates that the difference in van der Waals dispersive contribution is not sufficient to determine the binding specificity of DMH1 among the three kinases.

In contrast to the van der Waals dispersive contributions, which consistently favor the binding process, the contribution of electrostatic interactions is only favorable in ALK2 (Table 1). This indicates that the sum of favorable electrostatic interactions associated with hydrogen bonding and charge-charge (salt bridge) interactions established between DMH1 and ALK2 surpass the loss of water-DMH1 interactions in bulk solution. However, in ALK5 and VEGFR2, the sum of electrostatic interactions between ligand and binding site is not enough to compensate for the desolvation penalty of DMH1. Therefore, although the total binding free energy is dominated by the dispersive contribution, the electrostatic contribution is the key determinant responsible for the binding specificity of DMH1 to ALK2 over ALK5 and VEGFR2. In the following computational analysis section, we discuss what those key interactions are.

### Computational Analysis

#### Per-residue interaction energy reveals key amino acids dominating ALK2 specificity

**Fig 3:**
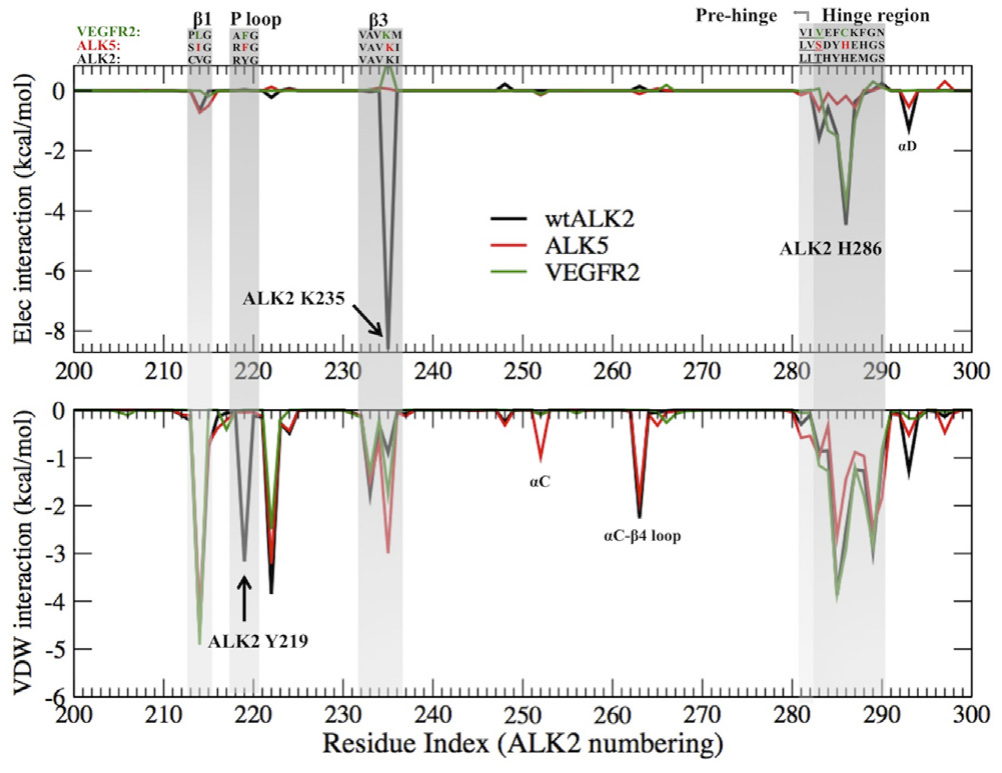
Per-residue interaction with DMH1 in wtALK2 (black), ALK5 (red) and VEGFR2 (green), averaged from the last 2 ns of trajectory. Top: electrostatic interaction. Bottom: van der Waals interaction. The three protein sequences are aligned by homology and the numbering is that of ALK2. The rest of the residues that have no interaction with DMH1 are not shown.

The per-residue electrostatic interaction between DMH1 with ALK2, ALK5, and VEGFR2 shows clearly that the most favorable electrostatic interaction between DMH1 and ALK2 Lys235 is missing in ALK5 and VEGFR2 (Fig 3 top). It is consistent with the fact that a direct hydrogen bond between the Lys235 side chain and the N atom on the quinoline moiety of DMH1 was observed in 20% of the whole simulation time in ALK2, but in 0% with the corresponding Lys232 in ALK5 and Lys868 in VEGFR2. In addition, the favorable electrostatic interactions at the hinge region are much more significant in ALK2 than in ALK5. This is because at the ALK2 hinge region His286 forms a stable hydrogen bond with the N1 atom of the DMH1 pyrazole ring (Fig 1). This conserved hydrogen bond has been observed in the crystal structures of ALK2-dorsomorphin and ALK2-LDN193189 complexes [10, 20]. The van der Waals interactions are similar among ALK2, ALK5 and VEGFR2, except that the favorable van der Waals interaction between DMH1 and Tyr219 in ALK2 is absent in ALK5 and VEGFR2 (see discussion on Tyr219 below).

#### P-loop Tyr219 in ALK2 significantly contributes to binding affinity and selectivity

**Fig 4:**
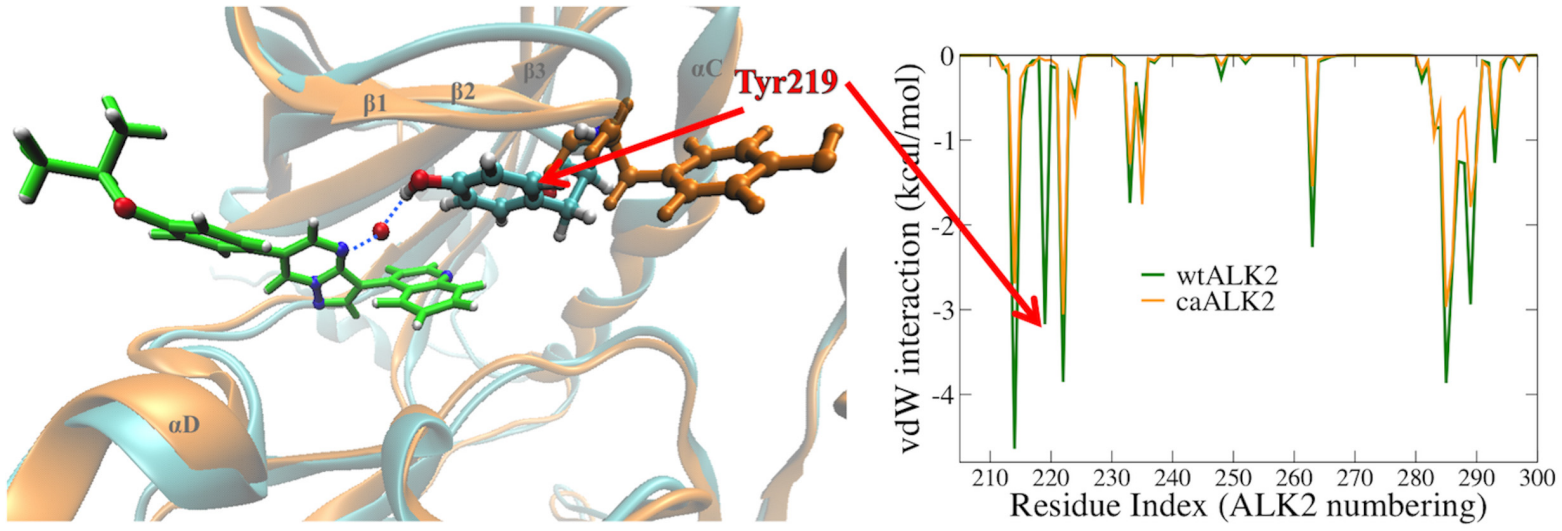
**Left:** Superposition of wtALK2 (cyan) and caALK2 (orange) backbone in cartoon. P-loop Tyr219 is shown in sticks. The ligand DMH1 is shown in light green. Tyr219 in caALK2 (orange) is pointing away from DMH1. Tyr219 in wtALK2 (cyan) is pointing towards the ligand and forms a water-mediated hydrogen bond with N4 on the pyrimidine moiety of DMH1. **Right:** van der Waals interaction energies in kcal/mol between each ALK2 residues and DMH1: wtALK2 in green, caALK2 in orange.

**Fig 5:**
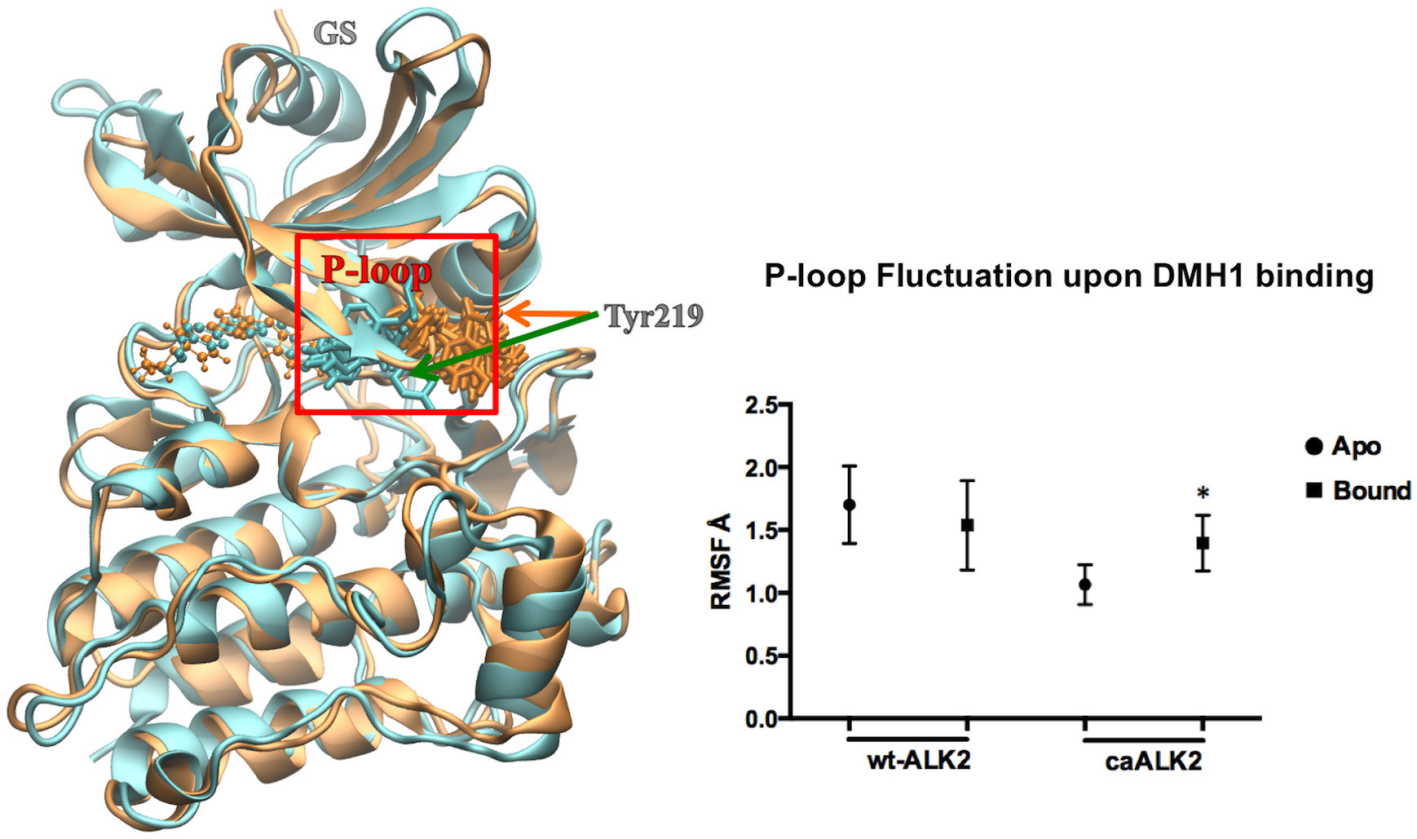
**Left:** Superposition of wtALK2 (cyan) and caALK2 (orange) backbone in cartoon. P-loop Tyr219 is shown in sticks with overlapped snapshots from the last 2 ns of simulation. DMH1 atoms are shown as small spheres. **Right:** RMSF of the P-loop in the unbound (apo) and DMH1 bound states. Statistical difference is represented by an * above the bound conformation; * = P < 0.05.

During our investigation of ALK2, we first used the caALK2 crystal structure (PDB ID 3Q4U). Although the docking score (estimated binding free energy) of DMH1 agrees well with the experimental value of -9.6 kcal/mol, the FEP/H-REMD calculated binding free energy is -6.2 kcal/mol (Table 1). When we used the wtALK2 crystal structure (PDB ID 3H9R), the binding free energy is -8.5 kcal/mol. Interestingly, the binding poses of DMH1 in wtALK2 and caALK2 are nearly identical. The averaged van der Waals interactions between the binding site and DMH1 also show a similar pattern, except that a favorable P-loop (also termed phosphate-binding loop) Tyr219 peak in wtALK2 is completely missing in caALK2 (Fig 4). Dynamic trajectories showed Tyr219 in wtALK2 always pointing inwards (Tyr219-in), forming favorable hydrophobic contacts with the quinoline moiety of DMH1 and a water-mediated hydrogen bond with N4 on the pyrimidine moiety 25% of simulation time (Fig 4). In contrast, Tyr219 in caALK2 is pointing outwards away from the ligand throughout the simulation (Fig 5 left). In addition, the root mean square fluctuation (RMSF) of the P-loop indicated that its fluctuation did not change upon DMH1 binding to wtALK2; whereas, DMH1 binding to caALK2 increased RMSF (Fig 5 right). Thus, the differences in both the interaction energy and the P-loop fluctuation confirm that the favorable interaction between DMH1 and Tyr219 can only be established when Tyr219 is pointing inward as in the wtALK2.

Our structural analysis and PDB database search suggest that this conformational difference in the P-loop is unlikely to be caused by a single mutation in Q207D. In fact, another Q207D ALK2 crystal structure (PDB ID: 4BGG) has the same Tyr219-in conformation as in wtALK2. Different inhibitors in the binding site may also contribute to this difference in the Tyr219 orientation among crystal structures. Recently there was a study about the effect of different inhibitors on the orientation of P-loop Phe632 in PRK1 kinase crystal structure (PDB ID: 4OTI) [58]. Other factors such as the presence of the GS domain on top of kinase N-lobe motif may also affect the P-loop conformation. Noticeably, four currently available crystal structures of Q207D caALK2 (PDB ID: 3Q4U, 4BGG [18], 3OOM and 3MTF [20]) all have the truncated GS domain, which may remove potential inhibitory interactions in the crystal structure of the protein, thus change the P-loop conformation. The caALK2 structure used here may represent a metastable state of ALK2 inactive conformation. In conclusion, the current analysis reveals quantitatively the crucial impact of the P-loop Tyr219 residue on the inhibitor binding affinity and selectivity, which indeed deserves special attention during structure-based ALK2 inhibitor design.

#### The difference in the pre-hinge region affects the DMH1 binding pose in ALK2 and ALK5

**Fig 6:**
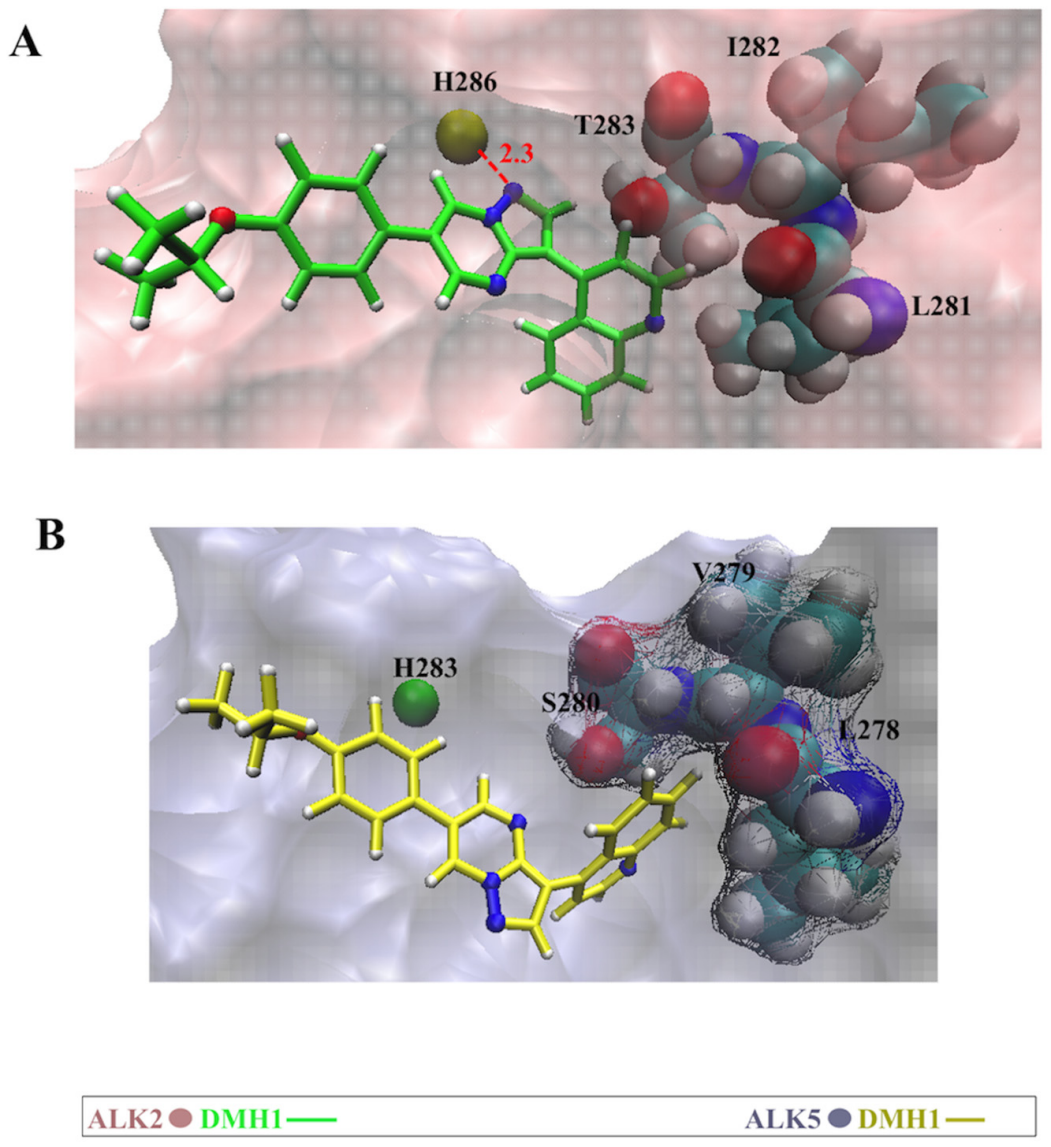
Binding conformations of DMH1 in ALK2 (top) and ALK5 (bottom) from molecular dynamics simulations. The conserved triad of amino acids consists of the gatekeeper and two pre-hinge residues shown in VDW mode. The rest of the protein is shown in surface mode.

As previously described, the docking pose of DMH1 in ALK2 shares similar features with the ALK2-LDN193189 complex crystal structure, in which the pyrazolo[1,5-**a**]pyrimidine moiety of DMH1 faces the hinge region and forms a direct hydrogen bond with His286 in ALK2 (Fig 6A). This hydrogen bond is also seen in other crystal structures of BMPRI kinases, ALK6 (PDB ID: 3MDY) and ALK1 (PDB ID: 3MY0), in complex with LDN193189. However this favorable electrostatic interaction is absent between DMH1 and ALK5 (Fig 3). In search of a possible explanation, we compared the sequence conservation among three families of kinases (Table 2). We found that BMPRI kinases (ALK1, 2, 3 and 6) share a conserved amino acid triad Leu281, Ile282 and Thr283 (ALK2 numbering) adjacent to the ATP binding site, which we denote the “pre-hinge” region. Thr283 is known as the “gatekeeper” as it blocks access of ligands to a hydrophobic pocket next to the site of ATP binding [26]. In TGF-β kinases (ALK4, ALK5, ALK7), this pre-hinge triad consists instead of a conserved sequence Leu278, Val279 and Ser280 (ALK5 numbering) [14, 19, 59]. In our simulation, this less bulky pre-hinge triad in ALK5 allows the DMH1 quinoline ring to reach deeper within this hydrophobic region (Fig 6B). This binding pose shift results in the loss of two major favorable electrostatic interactions between DMH1 and His283 and Lys232 in ALK5. Noticeably, ALK4, which has the same pre-hinge triad as ALK5, also does not bind DMH1. On the other hand, crystal structures of ALK5 with the potent ALK5 inhibitors SB431542 (PDB ID 3TZM), GW855857 (PDB ID 3HMM), compound 19 (PDB ID 2WOU), and indolinone (PDB ID 2X7O) [26, 60–62] indicate that their binding is associated with two hydrogen bonds, one of which must be with the hinge region His283. Therefore, our model can explain why DMH1 is not a potent inhibitor of ALK5.

**Table 2:** Hinge region sequences of ALK1-7 and the IC_50_ values of LDN193189 and DMH1.

*Family*	*Kinase* ^*a*^	*Pre-hinge*	*Gatekeeper*	*Hinge*	*LDN 193189 IC*_*50*_ *(nM)*	*DMH1 IC*_*50*_ *(nM)*
**BMPR-I**	ALK1	LI	T	HYHEHGS	13.3	27
	ALK2	LI	T	HYHEMGS	40.7	107.9
	ALK3	LI	T	DYHENGS	<5	<5
	ALK6	LI	T	DYHENGS	60	47.6
**TGF-βR**	ALK4	LV	S	DYHEHGS	1825	9622
	ALK5	LV	S	DYHEHGS	565	>100,000
	ALK7	LV	S	EYHEQGS	N/A	N/A

^***a***^ IC_50_ values are taken from reference [14] for each kinase.

#### Solvent exposed piperazine ring makes LDN193189 a more potent inhibitor of ALK5 than DMH1

**Fig 7:**
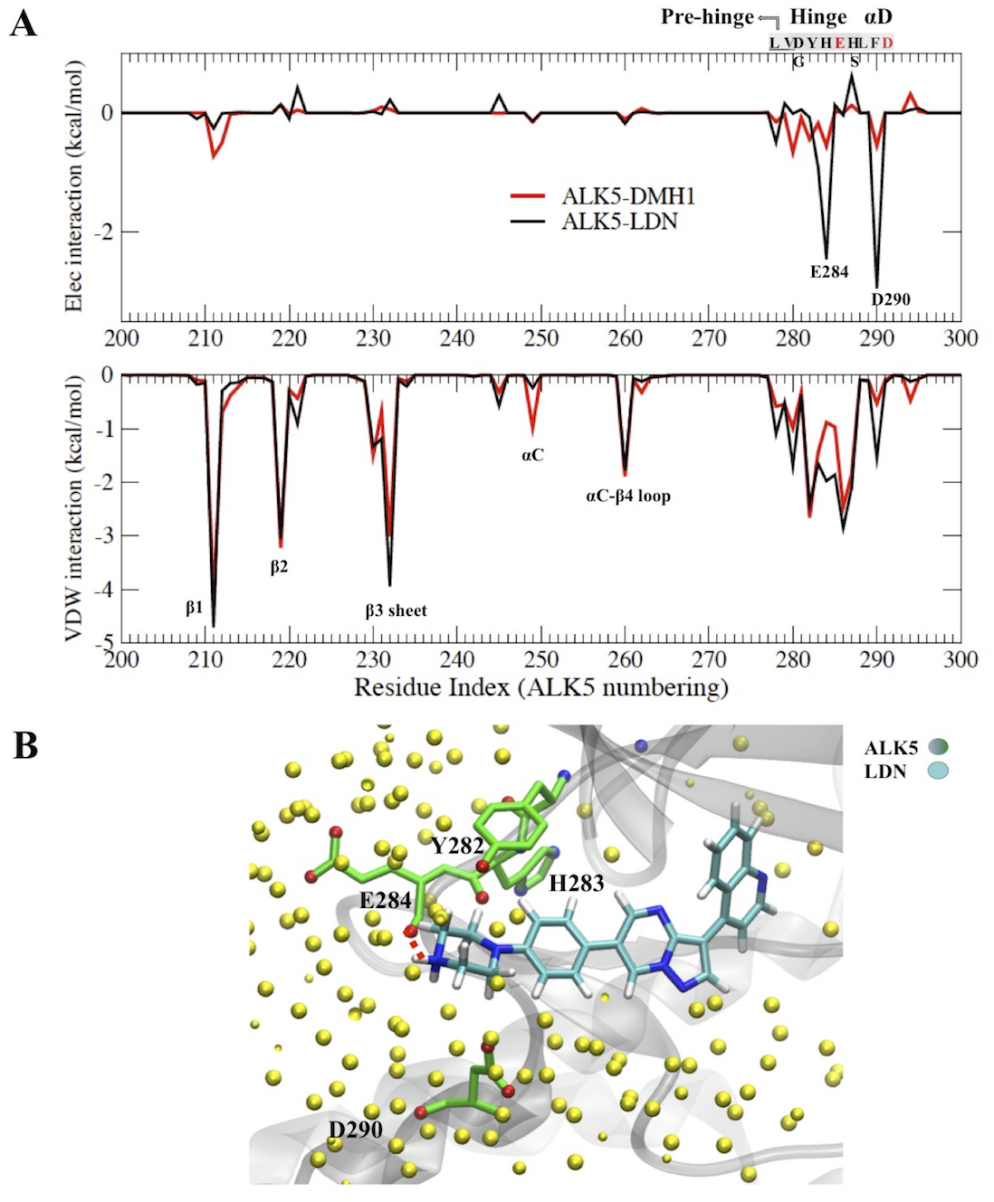
**A.** Residue decomposed electrostatic interactions (top) and van der Waals interaction (bottom) between LDN193189 and ALK5 (black line), compared with DMH1 (red line). **B.** Residues in ALK5 that contribute favorable electrostatic or van der Waals interactions with the LDN193189 piperazine moiety are highlighted in green. A hydrogen bond between the LDN193189 protonated N and Glu284 backbone is shown as a red dotted line. Water molecules within 8 Å of LDN193189 are shown as yellow spheres.

Compound LDN193189 is a close analog of DMH1 (Fig 1). The piperazine ring in LDN193189 [16] was designed to replace the solvent exposed moiety of dorsomorphin in order to improve the solubility and metabolic stability by avoiding the phase I O-dealkylation metabolic pathway. However, LDN193189 turned out to be a more potent inhibitor of ALK2 and also ALK5 compared with DMH1 (Table 2). In order to explain the difference between DMH1 and LDN193189 in their interaction with ALK5, we used the fully equilibrated ALK5-DMH1 conformation, and replaced DMH1 with LDN193189 by substituting the isopropoxy moiety of DMH1 with a piperazine ring using the Molecular Operating Environment (MOE) program [63]. The new ALK5-LDN193189 complex was then solvated in explicit solvent and submitted for further minimization and molecular dynamics simulation. The RMSD and the distance of center of mass between ligand and receptor show that LDN193189 quickly reaches equilibrium in ALK5 within 18 ns of simulation (S5 Fig). The equilibrated binding poses of LDN193189 and DMH1 are essentially the same, since the two molecules are highly similar. The average per-residue electrostatic interaction from the last 6 ns reveals clearly a more favorable electrostatic interaction between LDN193189 and ALK5 Glu284 (hinge region) and Asp290 (αD helix) residues (Fig 7A). Hydrogen bonding analysis indicates that the protonated piperazine ring of LDN193189 forms a hydrogen bond with Glu284 40% of the simulation time (Fig 7B). The van der Waals interaction between ALK5 and LDN193189 is also stronger than with DMH1 at the hinge region (Fig 7A). Therefore, our model illustrates that the solvent exposed R2 group in dorsomorphin analogs (Fig 1) also plays an important role in binding selectivity. This group can be modified to manipulate the binding selectivity between ALK isoforms.

#### Electrostatic interaction between DMH1 and VEGFR2 is not sufficient to establish a strong binding

Our FEP/H-REMD calculations demonstrate that DMH1 has very low binding affinity toward both VEGFR2 DFG-in and DFG-out conformations (binding free energy of -3.4 and -1.7 kcal/mol, respectively). The DMH1 pose (3) in VEGFR2-in/out from docking is similar to LDN193189 in the ALK2 crystal structure (Fig 2). However, the molecular dynamics simulation in the fully solvated system brought to light the deviation of DMH1 from its original docked pose in VEGFR2 within 10 ns. The most relevant ligand motion occurs by the rotation of the quinoline ring when it binds to VEGFR2 (S6A Fig). Due to this deviation, DMH1 in VEGFR2, compared to that in ALK2, misses a major electrostatic interaction and hydrogen bond with Lys868 of the β3 strand. A survey of 28 x-ray crystal structures of VEGFR2-inhibitor complexes (see all PDB IDs [64]) also indicates that potent VEGFR2 inhibitors typically form two to three direct hydrogen bonds with Cys919 (hinge region) and/or Asp1046 (DFG) and occasionally Glu885 (αC helix). Compared to all the potent VEGFR2 inhibitors, the molecular dynamics-equilibrated DMH1 only forms one direct hydrogen bond with Cys919 (S6B and S6C Fig). In conclusion, both the positive electrostatic free energy component (Table 1) and the PDB database survey reveal that DMH1 does not establish the necessary favorable electrostatic interactions with VEGFR2.

#### Effect of DMH1 binding on the salt-bridge network and the A-loop

**Fig 8:**
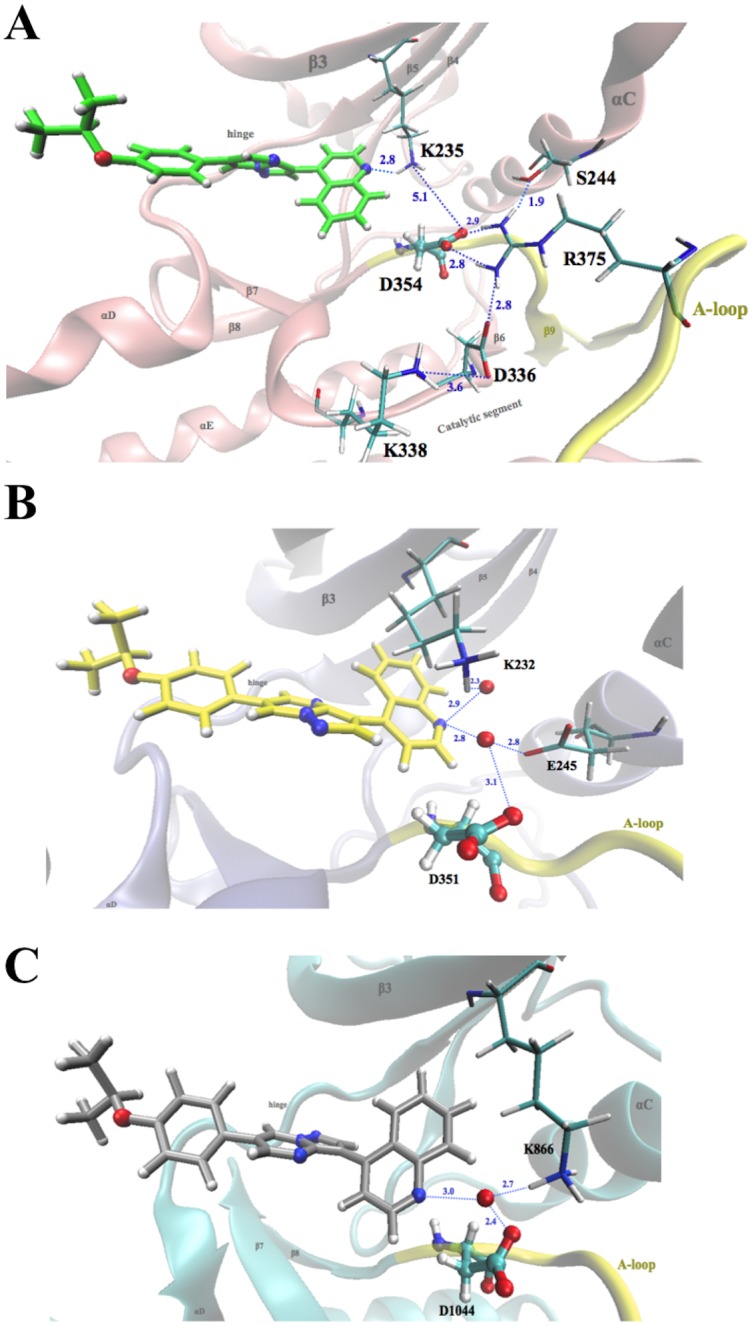
**A.** Hydrogen-bonding and salt-bridge network of the wtALK2-DMH1 complex. Protein backbone is partially shown. DMH1 and residues involved in the network are shown in stick with atoms colored by type: red, oxygen; blue, nitrogen; green, carbon; and white, hydrogen. A-loop backbone is shown in yellow. The average distance of each salt bridge and hydrogen bond is indicated. **B.** ALK5-DMH1 complex showing water molecule as red ball bridging two hydrogen bonds between Lys232 and Asp351 and the nitrogen atom on the quinolone ring of DMH1. **C.** VEGFR2-in conformation and numbering showing water molecule bridging two hydrogen bonds between Lys866 and Asp1044 and the nitrogen atom on the quinolone ring of DMH1.

Previous ALK2 crystal structures show that there are hydrogen-bonding and salt-bridge networks between Lys235 (β3 strand) and Asp354 (DLG motif), and between Arg375 (A-loop) and Ser244 (αC-helix), Asp336 (catalytic segment), and Asp354 [10]. Here, we analyze the dynamics of hydrogen bonding and salt bridges by calculating the probability of their occurrence (i.e., occupancy) in presence of DMH1 during the simulation. In ALK2, a direct hydrogen bond between DMH1 quinoline nitrogen and Lys235 side chain weakens the salt bridge between Lys235 in the β3 strand and Asp354 in the A-loop DLG-motif (Fig 8). As a result, the A-loop fluctuation in wtALK2 and caALK2 increases upon DMH1 binding (p = 0.0069 and p = 0.0174, respectively) (S7 Fig). In the binding models of ALK5 and VEGFR2 in the in and out conformations, DMH1 only forms water-mediated hydrogen bonds with Lys232 (β3 strand) and Asp351 (DLG motif) in ALK5, or Lys866 (β3 strand) and Asp1044 (DFG motif) in VEGFR2 in both in and out conformations (Fig 8B and 8C). The network of Lys232-DMH1-Asp351 water-mediated hydrogen bonding plus Lys232-Asp351 salt bridge (ALK5 numbering) stabilizes the A-loop compared to the unbound structure (p < 0.001 for ALK5 and VEGFR2-in, and p = 0.0468 for VEGFR2-out). Therefore, the dynamic analysis here emphasizes that the hydrogen bonding and salt bridge network near the ATP binding site are tightly tied. Weakening or strengthening a single hydrogen bond by a small molecule such as DMH1 is likely to have long distance effects on the strength of the hydrogen-bonding and salt-bridge network as well as the dynamics of the A-loop. This finding is consistent with the dynamically coupled allosteric network in Src kinase previously reported [65].

## Conclusions

Given the important roles of BMP signaling in embryogenesis and homeostasis, small molecules that specifically target BMPRIs are highly sought after. In recent years, BMP inhibitors including dorsomorphin, DMH1, LDN193189 and other analogs, have been developed to inhibit BMPRI subtype ALK2. However, the molecular mechanism underlying their binding selectivity between ALK2 and other structurally closely related kinases has remained unknown. In the present study, we used computational tools such as docking, molecular dynamics simulation and free energy calculations to address this issue. While our docking scores from AutoDock did not differentiate the binding selectivity of DMH1 among ALK2, ALK5 and VEGFR2, our FEP/H-REMD simulations successfully reproduced the fact that DMH1 only binds to ALK2, but not ALK5 and VEGFR2, in excellent agreement with experimental measurements. The free energy decomposition analysis showed that van der Waals dispersive interactions dominate the total binding affinity, but electrostatic interactions are largely responsible for DMH1 discrimination between ALK2/5 and VEGFR2. The per-residue interactions between the ligand and the kinases clearly revealed that the favorable electrostatic interaction with catalytic Lys235 and van der Waals interaction with the P-loop Tyr219 play critical roles in ALK2 binding specificity. A shift in the DMH1 binding pose in ALK5, mainly caused by the pre-hinge triad including gatekeeper Ser280 residue, results in the loss of several favorable interactions between the ligand and receptor. To understand the tighter binding of LDN193189 to ALK5, we performed molecular dynamics simulation of LDN193189 in ALK5 with explicit solvent. The simulation showed that the protonated piperazine ring on LDN193189 forms stable hydrogen bonds with Glu284 in ALK5. Our analysis provides the rationale for improving ALK2/ALK5 selectivity of LDN193189 analogs through modifying the solvent exposed group.

In summary, the current study reveals how small changes in the binding site residue type (e.g. pre-hinge region in ALK2 *vs*. ALK5) or residue conformation (e.g. Tyr219 in caALK2 *vs*. wtALK2), as well as small ligand modification (e.g. DMH1 *vs*. LDN193189) will cause distinct binding profiles and selectivity. It is, therefore, difficult to predict the binding specificity of small molecules in BMPI receptors solely based on the ligand-based structure-activity relationship or static binding information from rigid protein docking and crystal structures. In contrast, the computational methodology applied in this study takes into consideration local conformational changes as well as the effect of explicit solvent, representing a new way in understanding binding specificity of small molecule BMP inhibitors to their receptor kinases, which is critical for developing exclusively selective inhibitors for each subtype of BMPRI.

In terms of computational cost, each 1ns FEP/H-REMD simulation took approximately 5 hours real time (128 replicas running on 512 compute nodes in parallel on the Mira Blue Gene/Q supercomputer). The time-evolution of the absolute binding free energy plot suggests that even though it took 2–4 times longer to get a converged absolute binding free energy, the rank of the binding free energies among the three kinases is correct within 10 hours real time for each system. Therefore, the current study demonstrates that the FEP/H-REMD approach can serve as a robust method to validate the binding poses from virtual docking when the crystal structure of a ligand-receptor complex is not available. Furthermore, we show that molecular dynamics-based free energy simulation can explain and predict binding selectivities of BMP inhibitors among highly conserved ATP binding sites. Our computational approach presented here would play a significant role in the rational design of exclusively selective and potent BMP inhibitors.

## Supporting Information

S1 Fig Structure of ALK2 with labeled regions (Fig A). Structural alignment of ALK2 and ALK5 (Fig B). Structural alignment of ALK2 with VEGFR2-in and VEGFR2-out (Fig C).
(PDF)

S2 Fig The time evolutions of the RMSD of protein and ligand, as well as the center-of-mass distance between protein and ligand in each simulation system. (DOCX)

S3 Fig Thermodynamic cycle used to calculate the absolute binding free energy. (DOCX)

S4 Fig Progression of the total binding free energies of DMH1 in different systems with respect to the FEP/H-REMD simulation time (Fig A). Progression of the free energy components with respect to the coupling parameters (Fig B).
(DOCX)

S5 Fig The time evolutions of the RMSD of ALK5 and the distance between the center-of-mass of the LDN193189 and ALK5 during the equilibrium simulation. (DOCX)

S6 Fig The motion of DMH1 deviating from its original docked pose (Figure A). Alignment of VEGFR2-out DMH1 complex with compound-19 in PDB ID 3VO3 (Figure B). Alignment of VEGFR2-in DMH1 complex with compound 11-b in PDB ID 3CJG (Figure C).
(DOCX)

S7 Fig Fluctuation of the A-loop backbone upon DMH1 binding. (DOCX)

S1 Table Structures of BMP inhibitors and fold selectivity against ALK2 kinase. (DOCX)
