# Supplementary material for: Uncovering Molecular Bases Underlying Bone Morphogenetic Protein Receptor Inhibitor Selectivity
Source: PLoS One. 2015 Jul 2;10(7):e0132221. doi: 10.1371/journal.pone.0132221 (PMC4489870; doi:10.1371/journal.pone.0132221)
Supplement: S1 Fig — Structure of ALK2 with labeled regions (Fig A). Structural alignment of ALK2 and ALK5 (Fig B). Structural alignment of ALK2 with VEGFR2-in and VEGFR2-out (Fig C). (PDF) [file pone.0132221.s001.pdf]

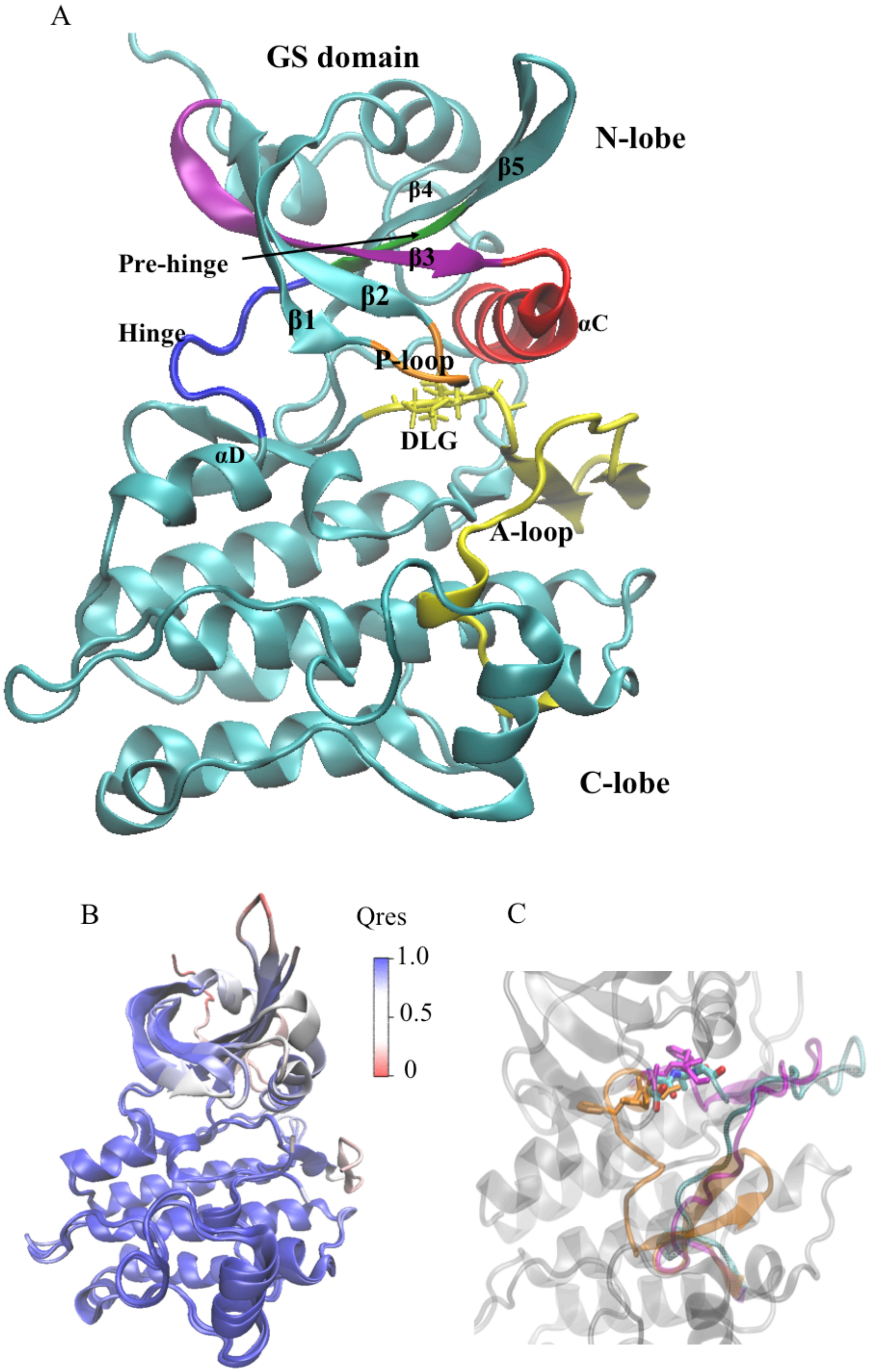

Figure S1. **A.** Structure of ALK2 with labeled regions. Highlighted in blue (hinge region), green (pre-hinge region) indicated by an arrow, yellow (A-loop), yellow sticks (DFG motif), red (αC helix), orange (P-loop), and magenta (β3 strand). **B.** Structural alignment of ALK2 (PDB ID: 3H9R) and ALK5 (PDB ID: 3TZM). The Q_{res} value is

shown on the aligned backbone using color scale. Q_{res}=1 (blue) indicates identical structures. **C.** Structural alignment of ALK2 (PDB ID: 3H9R) with VEGFR2-in (PDB ID: 3CJG) and VEGFR2-out (PDB ID: 3VO3). A-loop is highlighted in: cyan in ALK2, magenta in VEGFR2-in, and orange in VEGFR2-out. The GL/FG motif at the beginning of the A-loop is shown in sticks. The rest of the protein is only shown for ALK2 in grey. Co-crystallized ligands are not shown. The STAMP structural alignment is done using MultiSeq in VMD.
