# Supplementary material for: Uncovering Molecular Bases Underlying Bone Morphogenetic Protein Receptor Inhibitor Selectivity
Source: PLoS One. 2015 Jul 2;10(7):e0132221. doi: 10.1371/journal.pone.0132221 (PMC4489870; doi:10.1371/journal.pone.0132221)
Supplement: S2 Fig — (DOCX) [file pone.0132221.s002.docx]

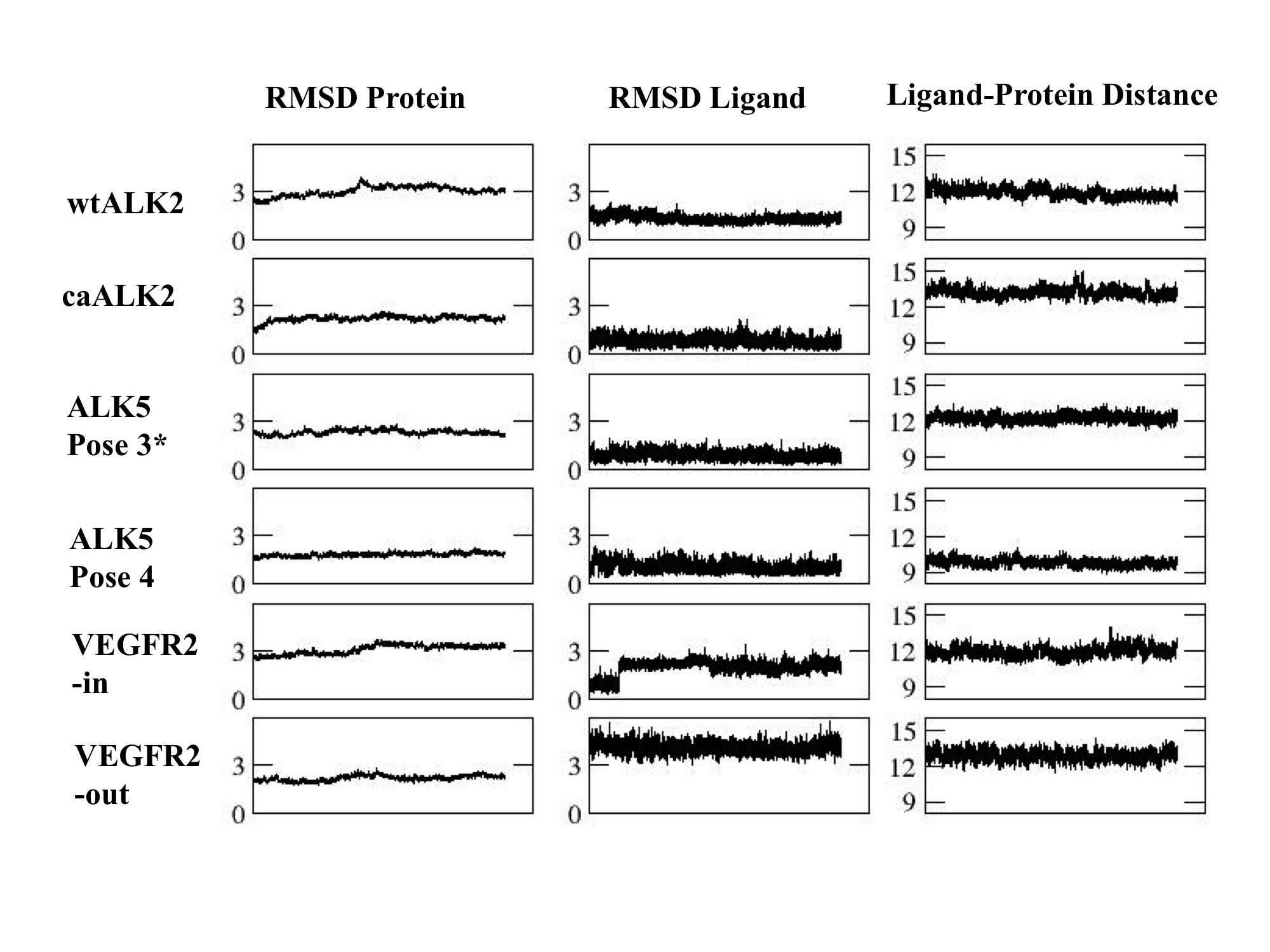


**Figure S2.** The time evolutions of the RMSD of protein backbone, DMH1 heavy atoms excluding solvent-exposed isopropoxy group, and the distance between the center-of-mass of the DMH1 and protein during the last 20 ns equilibrium simulations of all six systems before FEP/H-REMD calculations.
