# Supplementary material for: Uncovering Molecular Bases Underlying Bone Morphogenetic Protein Receptor Inhibitor Selectivity
Source: PLoS One. 2015 Jul 2;10(7):e0132221. doi: 10.1371/journal.pone.0132221 (PMC4489870; doi:10.1371/journal.pone.0132221)
Supplement: S3 Fig — (DOCX) [file pone.0132221.s003.docx]

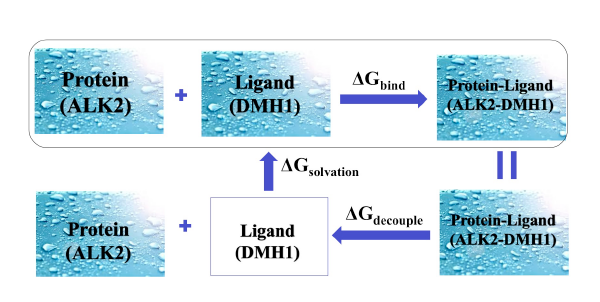


**Figure S3.** Thermodynamic cycle used to calculate the absolute binding free energy of DMH1 to ALK2. Solvent is represented as the blue background. Vacuum is represented as the white background.
