# Supplementary material for: Uncovering Molecular Bases Underlying Bone Morphogenetic Protein Receptor Inhibitor Selectivity
Source: PLoS One. 2015 Jul 2;10(7):e0132221. doi: 10.1371/journal.pone.0132221 (PMC4489870; doi:10.1371/journal.pone.0132221)
Supplement: S4 Fig — Progression of the total binding free energies of DMH1 in different systems with respect to the FEP/H-REMD simulation time (Fig A). Progression of the free energy components with respect to the coupling parameters (Fig B). (DOCX) [file pone.0132221.s004.docx]

**A**


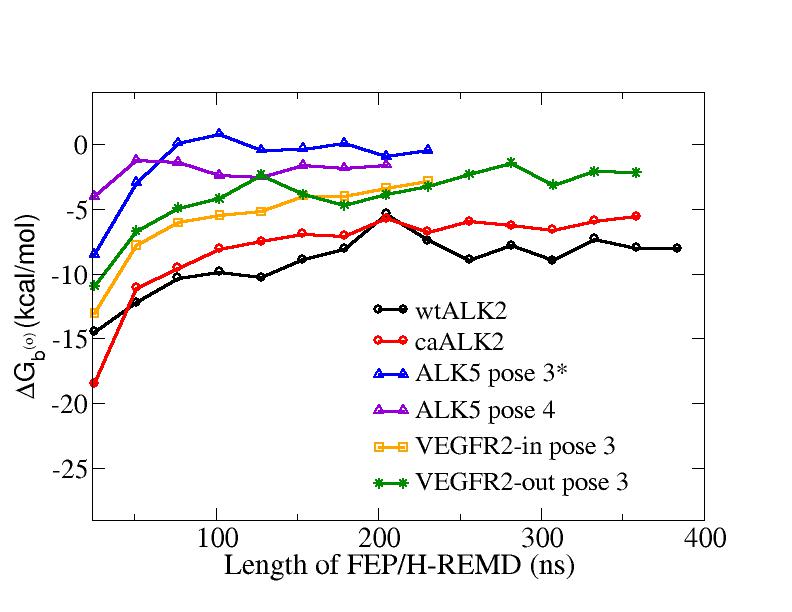


**B**


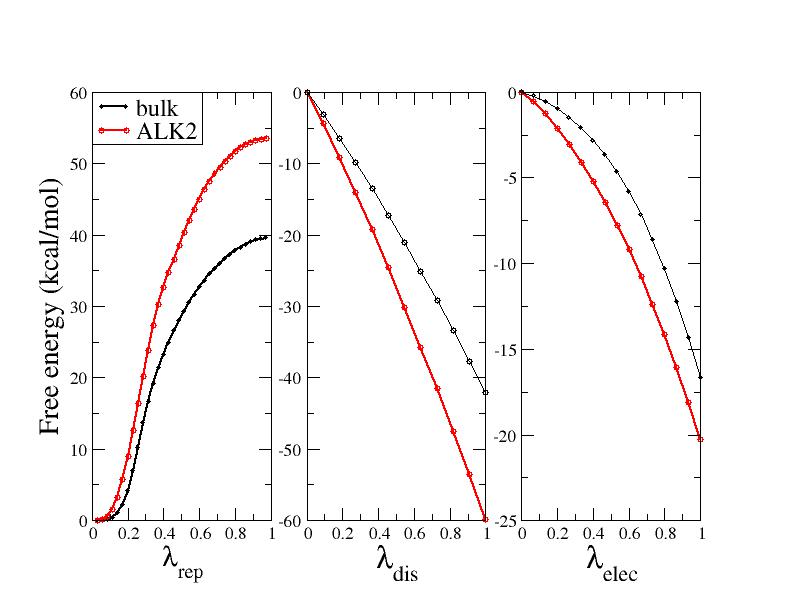


**Figure S4.** **A.** Progression of the total binding free energies of DMH1 in different systems with respect to the FEP/H-REMD simulation time (sum of all replicas). **B.** Progression of the free energy components with respect to the coupling parameters of repulsive, dispersive and electrostatic interaction for DMH1 in the binding site of wtALK2 (red) and in bulk (black).
