# Supplementary material for: Uncovering Molecular Bases Underlying Bone Morphogenetic Protein Receptor Inhibitor Selectivity
Source: PLoS One. 2015 Jul 2;10(7):e0132221. doi: 10.1371/journal.pone.0132221 (PMC4489870; doi:10.1371/journal.pone.0132221)
Supplement: S5 Fig — (DOCX) [file pone.0132221.s005.docx]

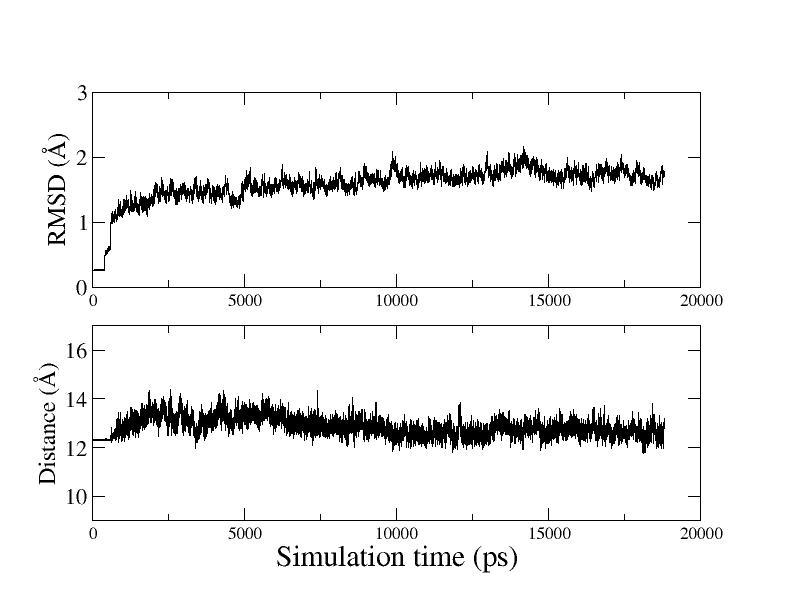


**Figure S5.** The time evolutions of the RMSD of ALK5 heavy atoms (top) and the distance between the center-of-mass of the LDN193189 and ALK5 (bottom) during the equilibrium simulation
