# Supplementary material for: Uncovering Molecular Bases Underlying Bone Morphogenetic Protein Receptor Inhibitor Selectivity
Source: PLoS One. 2015 Jul 2;10(7):e0132221. doi: 10.1371/journal.pone.0132221 (PMC4489870; doi:10.1371/journal.pone.0132221)
Supplement: S6 Fig — The motion of DMH1 deviating from its original docked pose (Figure A). Alignment of VEGFR2-out DMH1 complex with compound-19 in PDB ID 3VO3 (Figure B). Alignment of VEGFR2-in DMH1 complex with compound 11-b in PDB ID 3CJG (Figure C). (DOCX) [file pone.0132221.s006.docx]

**
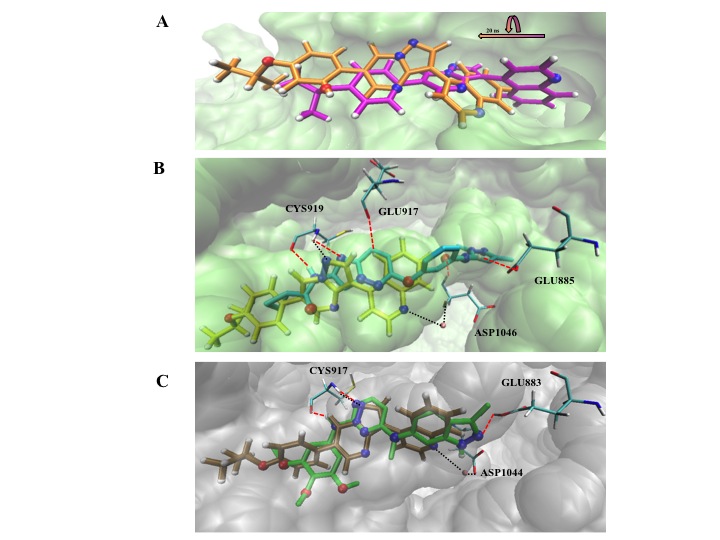
**

**Figure S6**. **A**. The motion of DMH1 (orange) deviating from its original docked pose (purple) within 10 ns of simulation. **B**. VEGFR2-out, in green surface, DMH1 in yellow, aligned with compound-19 in cyan (PDB ID: 3VO3). Hydrogen bonds are shown as red dashed line for Compound-19 and black dotted line for DMH1. Compound-19 forms two hydrogen bonds with Cys919 backbone amino and carboxyl groups, as well as hydrogen bonds with Glu917, Glu885, and Asp1046 **C**. VEGFR2-in, in gray surface, DMH1 in tan, aligned with compound 11-b in green (PDB ID: 3CJG). Compound 11-b forms direct hydrogen bonds with Cys917 backbone and Glu883 side chain. The post equilibrated DMH1, in both VEGFR2-in and out, only forms a single hydrogen bond with Cys919, and a water-mediated hydrogen bond with Asp1046 backbone carboxyl group (VEGFR2-out numbering).
