# Supplementary material for: Uncovering Molecular Bases Underlying Bone Morphogenetic Protein Receptor Inhibitor Selectivity
Source: PLoS One. 2015 Jul 2;10(7):e0132221. doi: 10.1371/journal.pone.0132221 (PMC4489870; doi:10.1371/journal.pone.0132221)
Supplement: S7 Fig — (DOCX) [file pone.0132221.s007.docx]

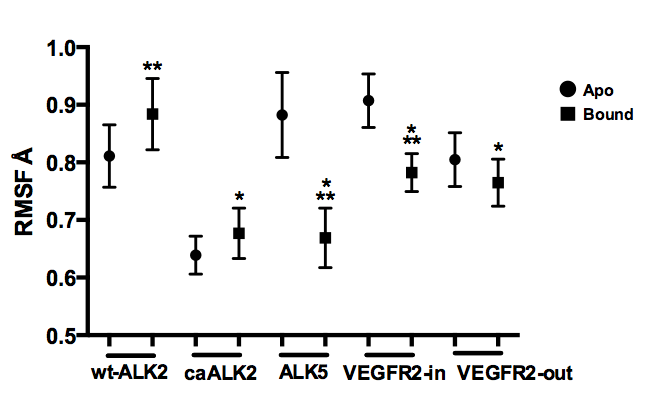


**Figure S7.** Fluctuation of the A-loop backbone upon DMH1 binding. Statistical differences between the apo and bound conformations are represented by an * above the bound conformation; * =P < 0.05, ** = p < 0.01, and *** = p < 0.001.
