# Supplementary material for: Uncovering Molecular Bases Underlying Bone Morphogenetic Protein Receptor Inhibitor Selectivity
Source: PLoS One. 2015 Jul 2;10(7):e0132221. doi: 10.1371/journal.pone.0132221 (PMC4489870; doi:10.1371/journal.pone.0132221)
Supplement: S1 Table — (DOCX) [file pone.0132221.s008.docx]

**Table S1:** Structures of BMP inhibitors and fold selectivity of the inhibitors against ALK2 kinase.

|  | BMPRI | | | | TGFβR | | VEGFR2 |
| --- | --- | --- | --- | --- | --- | --- | --- |
|  | ALK1 | ALK2 | ALK3 | ALK6 | ALK4 | ALK5 |  |
|  |  |  |  |  |  |  |  |
| Dorsomorphin^14^ | 1.6 | 1.0 | 1.4 | 3.5 | 381 | 253 | 0.3 |
| DMH1^14^ | 0.3 | 1.0 | <0.1 | 0.4 | 89 | >927 | >927 |
| LDN193189^14^ | 0.3 | 1.0 | <0.1 | 1.5 | 45 | 14 | 5.3 |
| LDN212854^19^ | 1.8 | 1.0 | 66 | N/A | 1641 | 7135 | 2154 |
| 7g^14^ | 1.4 | 1.0 | 338 | 307 | >3125 | >3125 | 616 |
| LDN214117^18^ | 1.0 | 1.0 | 43.4 | N/A | - | 111.1 | N/A |
